# Advances in immunology of obstructive sleep apnea: mechanistic insights, clinical impact, and therapeutic perspectives

**DOI:** 10.3389/fimmu.2025.1654450

**Published:** 2025-10-21

**Authors:** Na Dong, Hongmei Yue

**Affiliations:** ^1^ The First School of Clinical Medicine, Lanzhou University, Lanzhou, China; ^2^ Department of Respiratory and Critical Care Medicine, The First Hospital of Lanzhou University, Lanzhou, China

**Keywords:** obstructive sleep apnea, intermittent hypoxia, inflammation, immune dysregulation, CPAP, immunotherapy

## Abstract

Obstructive sleep apnea (OSA) drives immune dysregulation through its hallmark stressors—intermittent hypoxia (IH) and sleep fragmentation (SF). Beyond impaired sleep, OSA acts as a systemic inflammatory trigger that disrupts immune homeostasis and reshapes both innate and adaptive responses. Recent evidence shows that OSA activates hypoxia-inducible factor-1α (HIF-1α), NF-κB signaling, and the NLRP3 inflammasome, promoting chronic inflammation and immune-cell dysfunction. These alterations mechanistically contribute to OSA-associated cardiovascular disease, metabolic disorders, cognitive impairment, and tumor progression. Reframing OSA as an immune-modulating disorder highlights the need for diagnostics and therapies guided by immunology rather than airway management alone.

## 1 Introduction

Obstructive Sleep Apnea (OSA) is a sleep disorder characterized by recurrent upper airway obstruction, leading to apnea or hypoventilation, which leads to IH, nocturnal awakenings, daytime sleepiness, and cognitive impairment. Approximately 936 million people worldwide between the ages of 30 and 69 are affected by OSA, including about 176 million in China, corresponding to a prevalence rate of approximately 8.8% (1). As metabolic syndrome rates rise, the number of individuals suffering from OSA is also rising, making it a significant public health issue. Risk factors for OSA include obesity, age, anatomical features (e.g., large tongue and short, thick neck), family history, and conditions like hypertension and diabetes may increase the risk of OSA by influencing upper airway obstruction or sleep quality. The apnea-hypopnea index (AHI) is currently the main indicator for assessing the severity of OSA. An AHI of 5–15 events per hour is considered mild, 15–30 events per hour is moderate, and ≥30 severe. OSA’s pathophysiology involves IH, sleep architecture disruption, and abnormal sympathetic nervous system activation. This disorder not only affects sleep quality but also leads to various systemic health issues, including cardiovascular and metabolic conditions, neurocognitive dysfunction, and even multi-organ and multi-system dysfunction (2–8). The complex pathophysiological mechanisms associated with OSA are commonly linked to oxidative stress induced by IH, ongoing inflammatory cascades, molecular-level alterations, and increased sympathetic nervous activity (9). Recent research has increasingly explored the intricate relationship between OSA and the immune system, which contributes to the elevated risk of chronic inflammation, cardiovascular diseases, metabolic syndrome, neurocognitive disorders, and other related conditions. The involvement of immune responses in OSA has become more prominent. Furthermore, the bidirectional relationship between immune dysregulation and OSA also impacts disease progression and management.

**Figure 1:**
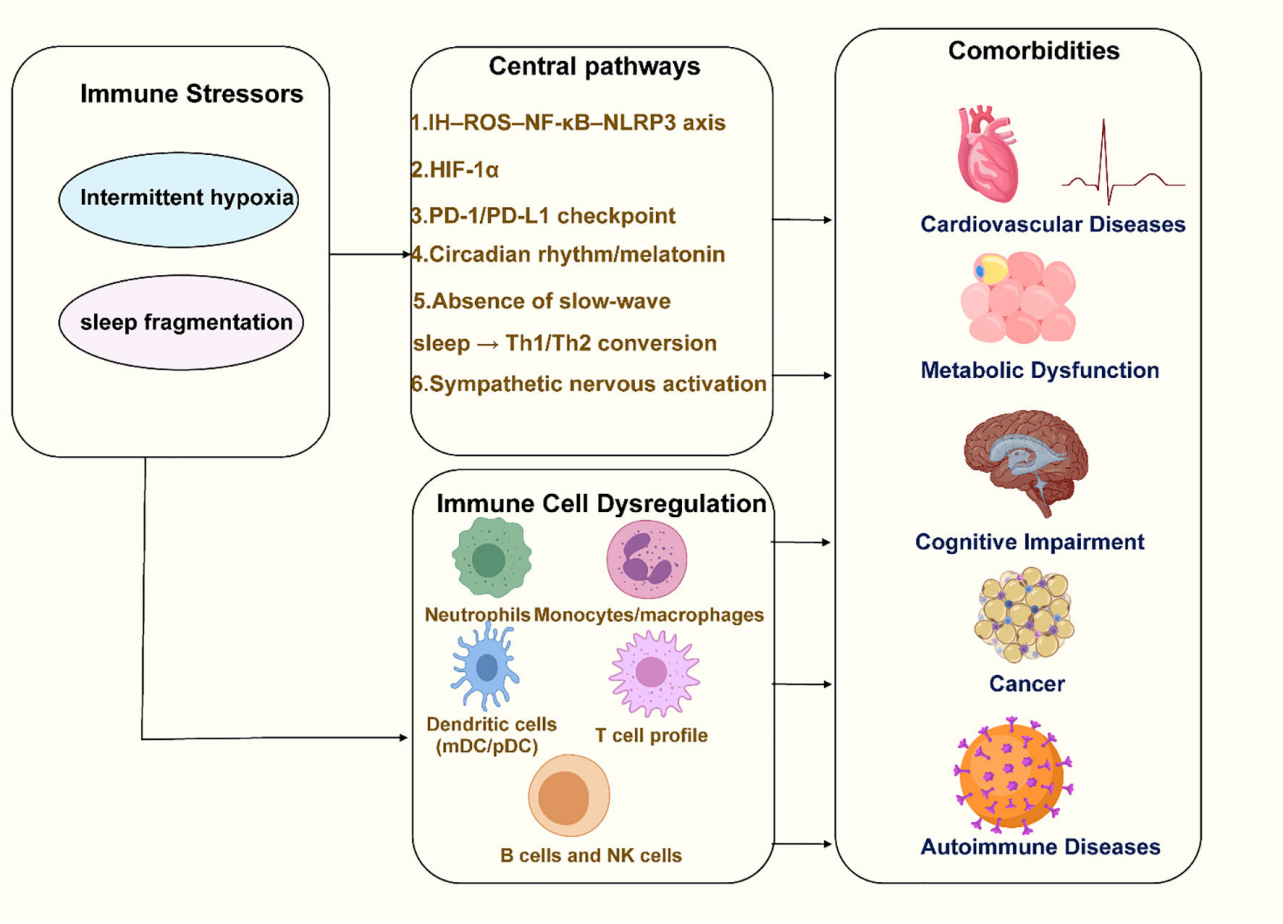
Overview of the pathophysiological connections between OSA and immune dysfunction. Created with MedPeer (medpeer.cn). Icons adapted and used under an institutional license for academic publication.

We systematically searched the PubMed, Embase, and Web of Science Core Collection databases for literature published between January 1, 1990, and January 30, 2025. The search terms combined Medical Subject Headings (MeSH) with free-text keywords, including “Obstructive Sleep Apnea” OR “OSA,” “Intermittent Hypoxia” OR “IH,” “Sleep Fragmentation” OR “SF,” “Immune” OR “Inflammation” OR “Immune Dysregulation” OR “Immune Response,” “Cardiovascular” OR “Metabolic” OR “Cognitive” OR “Cancer” OR “Comorbidity,” and “CPAP” OR “Immunotherapy.” We used Boolean operators (AND/OR) to combine the search terms, for example: “Obstructive Sleep Apnea” OR OSA AND (“Immune” OR “Inflammation” OR “Immune Dysregulation”) AND (“Cardiovascular” OR “Metabolic” OR “Cognitive” OR “Cancer” OR “Comorbidity”). Only peer-reviewed English-language articles, including original research, clinical studies, and relevant reviews, were included. Additionally, we manually searched the reference lists of the included articles to further identify potentially relevant studies. This review aims to examine the pathophysiological connections between OSA and immune dysfunction, highlighting the immune system’s critical role in OSA-related comorbidities. A better understanding of the relationship between OSA and immune responses will help clinicians manage patients more effectively, support the development of new treatments, provide more comprehensive treatment strategies for OSA patients. **Figure 1** provides an overall overview of the review’s content, illustrating the key pathophysiological connections between OSA and immune dysfunction, as well as the role of the immune system in OSA-related comorbidities.

## 2 Immune stressors induced by OSA

**Figure 2:**
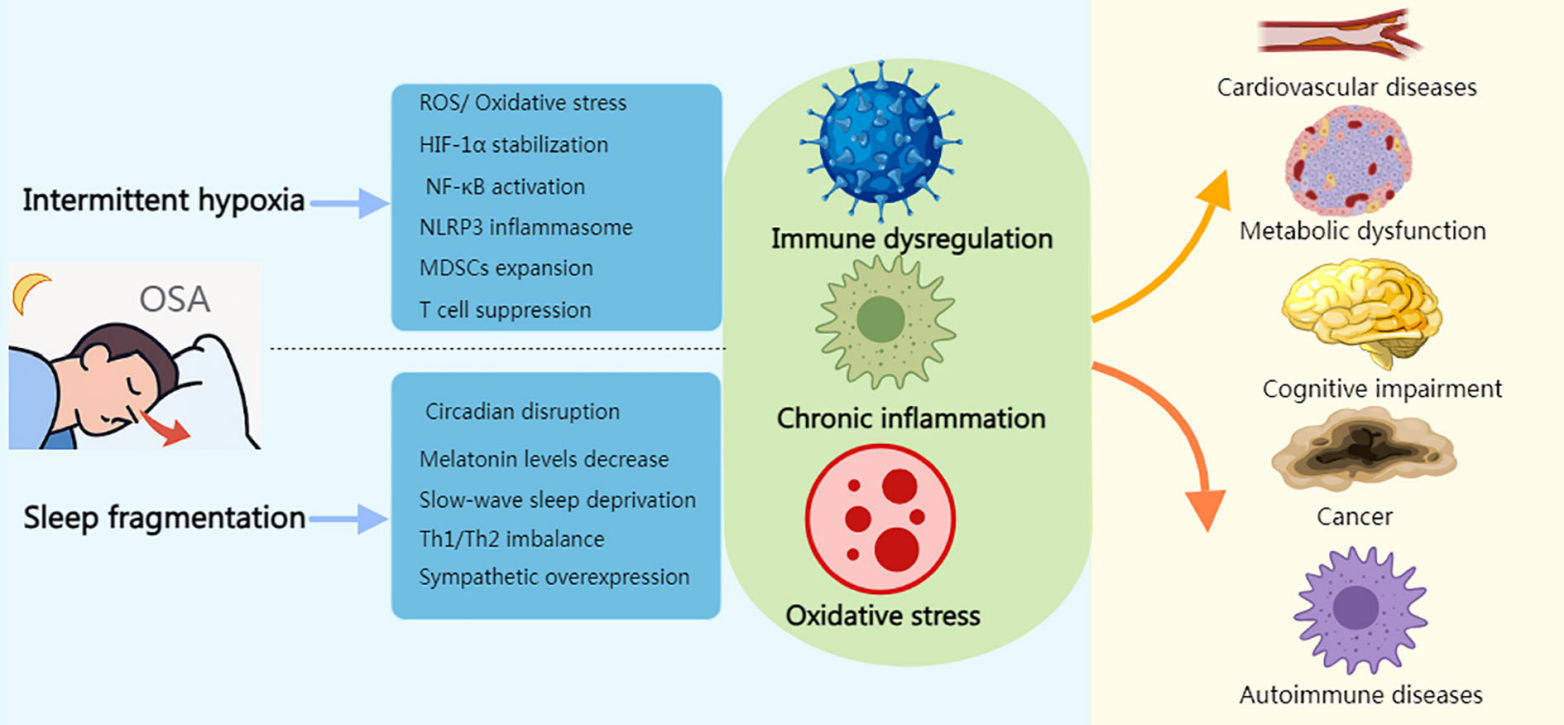
Immune stress response induced by OSA. Created with MedPeer (medpeer.cn). Icons adapted and used under an institutional license for academic publication.

Immune stressors induced by OSA refer to key stimuli triggered directly by the pathophysiological processes of OSA that activate the immune system. These stressors drive chronic inflammation and immune dysregulation through various molecular and cellular mechanisms, becoming central contributors to OSA-related comorbidities (**Figure 2**). The following are the major immune stressors and their mechanisms of action:

### 2.1 Intermittent hypoxia

#### 2.1.1 IH–ROS–NF-κB–NLRP3 axis

IH induced by OSA activates oxidative stress, metabolic, and immune pathways, leading to cumulative molecular and cellular damage, ultimately causing dysfunction and cell death (10). IH suppresses antioxidant defenses, raises ROS, and activates pro-inflammatory transcription factors such as NF-κB, creating a vicious cycle in which ROS both damages macromolecules and amplifies inflammation (11, 12). Phagocyte respiratory bursts and mitochondria-derived injury further increase ROS, reinforcing this feedback loop (13–15). In OSA patients, the alternating hypoxia and reoxygenation exacerbate ROS accumulation, leading to ATP depletion, calcium homeostasis disruption, and enhanced synthesis of pro-inflammatory factors and nitric oxide, impairing immune function (16). The NLRP3 inflammasome, activated by ROS, plays a central role in this cycle by promoting iNKT -1β maturation and triggering innate immunity (17). In severe OSA, NLRP3 activity is elevated, correlating with the AHI and hypoxia index. *In vitro*, OSA plasma enhances NLRP3 expression under both normoxic and hypoxic conditions, while oxLDL under IH further activates NLRP3 and IL-1β production (18, 19). Chronic IH upregulates NLRP3, and genetic or pharmacological inhibition reduces inflammation and oxidative stress in tissues (19–21). NLRP3-/- mice show reduced IL-1β secretion under IH, confirming the critical role of NLRP3 in IH-mediated inflammation (21, 22). This establishes the IH–ROS–NF-κB–NLRP3 axis, linking oxidative stress and inflammation in OSA-related pathogenesis (see Section 3.7 for related phenotypes and clinical associations).

#### 2.1.2 HIF-1α-mediated metabolic reprogramming and immune suppression

Hypoxia-inducible factor 1 (HIF-1) is a transcription factor made up of α and β subunits, where HIF-1β is stably expressed in the nucleus and is not regulated by oxygen concentration, whereas HIF-1α is found in the cytoplasm, and its stability is highly dependent on oxygen levels. Under normoxic conditions, HIF-1α is rapidly degraded, but under hypoxic conditions, it accumulates and translocates to the nucleus, where it binds to HIF-1β to form a transcriptionally active HIF-1 complex, which then initiates the expression of downstream target genes (23). A study by Xie et al. observed differences in the immune cell response to hypoxia in the peripheral blood of OSA patients, with the response intensity being positively correlated to the level of hypoxia (24). Further mechanistic studies revealed that IH can mediate significant immunosuppressive effects through multiple HIF-1α-dependent signaling pathways, including inducing lactate accumulation and metabolic pathway remodeling in the tumor immune microenvironment (TIME), inhibiting T cell proliferation and cytokine production, weakening T cell infiltration into tumor tissues, while promoting the expansion of myeloid-derived suppressor cells (MDSCs) and suppressing the antitumor functions of CD8^+^ T cells and natural killer (NK) cells (25).

#### 2.1.3 PD-1/PD-L1 checkpoint

Programmed cell death protein-1 (PD-1) and its ligand programmed death-ligand 1(PD-L1) constitute a key inhibitory axis that maintains T cell quiescence (26). HIF-1α can bind to the hypoxia response element in the PD-L1 promoter and directly regulate its transcriptional expression (27). Polasky et al. found that PD-L1 expression on peripheral monocytes and PD-1 expression on CD8^+^ T cells are significantly elevated in OSA patients (28) (see Section 3.8 for related phenotypes and clinical associations).

### 2.2 Sleep fragmentation

#### 2.2.1 Circadian rhythm/melatonin pathway

Serum melatonin levels progressively decrease with the severity of OSA (29). Melatonin exerts its anticancer potential through various mechanisms, including inhibiting cell proliferation, scavenging ROS, promoting apoptosis, antagonizing estrogen effects, and suppressing angiogenesis (30). Additionally, it contributes to immune regulation and anti-inflammatory effects by suppressing the NF-κB/NLRP3 inflammasome pathway and activating T cells, B cells, and macrophages, thereby further enhancing its antitumor effects (31).

#### 2.2.2 Absence of slow-wave sleep → Th1/Th2 conversion

Sleep is a key regulator of endocrine, metabolic, and immune homeostasis; its disruption accelerates the development and progression of chronic disease (32). Regular sleep preserves immune integrity and defenses against pathogens and inflammation, whereas circadian misalignment or poor sleep quality disrupts immune balance and elevates infection and inflammation risk (33, 34). In OSA, prolonged sleep fragmentation (SF) weakens immune defense and amplifies systemic inflammation; together with intermittent hypoxia (IH), SF synergistically triggers and sustains inflammation—the core pathological feature of OSA (35, 36). Physiologically, early-night slow-wave sleep (SWS) features nadir cortisol and peaks in growth hormone, prolactin, and aldosterone, a milieu that supports Th1-type responses and antimicrobial defense (37). However, slow-wave activity (SWA) is significantly suppressed in moderate–severe OSA and reduced with abnormal dissipation even in mild OSA (38, 39). Loss of SWS also raises cerebrospinal fluid β-amyloid (Aβ), linking sleep disruption to cognitive impairment (40, 41). Experimentally, sleep deprivation shifts immunity from Th1 to Th2 dominance, and older adults with insufficient SWS show a similar Th2 bias—changes that compromise anti-infective and antitumor surveillance (42–44).

#### 2.2.3 Sympathetic nervous activation

Activation of the sympathetic nervous system can inhibit the transcription of type I interferons (IFN-α/β) and their response genes, thereby weakening antiviral immunity (45). β-adrenergic receptor signaling can reduce T cell antitumor functions and has been shown *in vitro* to suppress Th1 responses while promoting Th2 responses (46). However, the causal relationship between SF-related immune phenotypes and sympathetic nervous system activation still requires further validation.

## 3 Immune cell dysregulation and regulation in OSA (phenotypes and evidence)

### 3.1 Neutrophils

The Neutrophil-to-Lymphocyte Ratio (NLR) is a key indicator of inflammation. Studies have shown that NLR levels in OSA patients are significantly higher than in healthy populations and are positively correlated with OSA severity. Additionally, NLR has been confirmed to have an independent association with coronary artery disease (47). Notably, in OSA patients treated with continuous positive airway pressure (CPAP), NLR values significantly decrease (48). This may be related to the activation and degranulation of neutrophils in the peripheral blood of OSA patients (49). In clinical practice, the reduction in NLR and its consistency depend on the quality of treatment: when compliance is adequate and residual events are well controlled, the decline is more significant and persistent. However, when compliance is poor, the follow-up period is short, or hypoxia persists, the changes in NLR are often not obvious. Residual heterogeneity may also stem from differences in research design, the composition of the subject population (especially the strong modifying effect of obesity/visceral fat on myeloid inflammation), concurrent infection or medication, and sampling/analysis variations, etc. Overall, the existing evidence supports that CPAP has an anti-inflammatory effect in reducing NLR. Meanwhile, it is suggested that when interpreting NLR, compliance indicators and hypoxia load endpoints (such as T90%, the lowest SaO_2_, ODI) should be combined, rather than relying solely on AHI.

### 3.2 Monocytes/macrophages (M1/M2 polarization)

Macrophages are widely distributed across tissues, and many originate from peripheral blood mononuclear cells (PBMCs) that migrate and differentiate into macrophages. Whether under physiological homeostasis or inflammatory stimuli, PBMCs can migrate to tissues and transform into macrophages (50). These blood-derived precursors of macrophages—monocytes—originate from hematopoietic stem cells during embryonic development and from the bone marrow in adults (51). Although most tissue-resident macrophages come from embryonic precursors, under specific conditions, circulating monocytes can also differentiate into tissue-resident macrophages with self-renewal capabilities (52). As key effector cells of the immune system, macrophages are responsible for clearing senescent cells, foreign particles, microorganisms, and tumor cells (53). Through their ability to phagocytose pathogens, recruit and regulate other immune cells, macrophages not only play a central role in host defense but also critically regulate the development of inflammation and degenerative diseases (54).

Studies have shown that following 4 weeks of continuous IH exposure, male mice exhibit a significant increase in pulmonary macrophages and ROS production (55). Macrophages are classified into two functionally distinct types: M1 (pro-inflammatory) and M2 (anti-inflammatory/repair) types (56). OSA promotes significant infiltration of M1 macrophages into subcutaneous adipose tissue and their accumulation within the aortic wall in chronic OSA mouse models, reflecting their critical role in systemic inflammation (57, 58). Mechanistically, OSA induces upregulation of HIF1α in atrial muscle cells through hypoxia/reoxygenation, which in turn enhances the expression of macrophage migration inhibitory factor (MIF). MIF binds to CD74 on macrophage surfaces, activating the NF-κB pathway and promoting M1 polarization. Polarized M1 macrophages release inflammatory cytokines, exacerbating atrial remodeling and increasing susceptibility to atrial fibrillation (AF). Macrophage depletion can reverse this process (59).

### 3.3 Dendritic cells (mDC/pDC)

Dendritic cells (DCs) play a crucial role in the immune system. However, there is still controversy regarding whether there is a reduction in DCs in OSA patients and the immune damage associated with this reduction. A study by Calati et al. reported a significant decrease in all DC subsets in the peripheral blood of OSA patients, particularly myeloid dendritic cells (mDCs) and plasmacytoid dendritic cells (pDCs). Furthermore, the reduction in DCs was concomitant with elevated levels of inflammatory cytokines and negatively correlated with IL-6 expression, thereby impairing the body’s ability to activate T cells (60). However, recent studies show no significant differences between the OSA group and healthy controls in the numbers of mDCs, pDCs, and the mDC/pDC ratio. Additionally, no significant correlation was found between the numbers of mDCs and pDCs and the AHI or the lowest oxygen saturation levels in OSA patients (24). This contradiction may stem from multiple factors: (i) Differences in research design and sample size (cross-sectional vs longitudinal, small sample single-center, and lack of parallel controls) lead to insufficient statistical power. (ii) Population heterogeneity (age, gender, smoking, and coexisting cardiometabolic diseases), especially obesity/visceral fat, has a significant modifying effect on the DC phenotype and circulation level; (iii) The methods of quantifying disease burden are inconsistent (only using AHI, without including indicators closer to hypoxia burden such as T90%, the lowest SaO_2_ or ODI), which may underestimate the DC changes associated with hypoxia; (iv) Treatment status and sampling time points (whether CPAP has been used previously, compliance, morning/evening blood collection, and circadian rhythm) can all affect DC count and activation phenotype. (v) Biological distribution and redistribution: Inflammation or tissue hypoxia can promote the migration of DC from peripheral blood to tissues (upper airway mucosa or adipose tissue), resulting in a decrease in peripheral blood count rather than a reduction in total volume. The above-mentioned methodological and biological heterogeneity jointly drive the contradictory conclusions about DC changes in the literature.

### 3.4 Abnormal T cell profile

OSA significantly impacts the immune system, particularly T cell populations. γδT cells and natural killer T cells (NKT), which link innate and adaptive immunity, show specific alterations in OSA patients. Studies reveal a reduction in perforin-positive CD3^+^γδT cells in peripheral blood, with their inhibitory effect increasing as oxygen saturation decreases (61). Animal models demonstrate initial activation of CD3^+^γδT cells in hypoxic environments, followed by a decrease. iNKT cells are reduced in OSA patients, with the reduction correlating with disease severity, while NKT-like cells increase in peripheral blood (62, 63). Additionally, changes are observed in CD4^+^ and CD8^+^ T cells, key cells of adaptive immunity. OSA patients show increased numbers of CD4^+^ and CD8^+^ T cells, with CD8^+^ T cells exhibiting an activated phenotype, especially subsets expressing natural killer receptors CD56 and CD16, which exhibit stronger cytotoxicity (62–64). OSA promotes a type 2 cytokine dominance in CD4^+^ and CD8^+^ T cells, enhancing their cytotoxicity and upregulating NK receptors, CD40L, perforin, and TNF-α (65, 66). In severe OSA, IH induces upregulation of PSGL-1 on T cells, impairing immune function and surveillance (67). CPAP treatment reduces the activation and cytotoxicity of these cells, with a decrease in total lymphocyte and CD4^+^ lymphocyte counts after 6 months, suggesting a reversal of immune activation in compliant patients (62, 65, 68). Studies on Th1/Th2 immune imbalance in OSA are inconsistent, with some showing Th1 cytokine activation and others indicating Th2 dominance linked to sleep disturbances and elevated catecholamines (65, 69).

### 3.5 B cells and NK cells

Studies show that both the proportion and number of B cells are significantly reduced in OSA patients, which is closely associated with metabolic disorders and obesity. B cell depletion appears to promote systemic inflammation (62). NK cells, essential for antiviral and antitumor responses, also play a critical role in maintaining the balance between innate and adaptive immunity (70, 71). In non-obese OSA patients, NK cell counts and IFN-γ levels are significantly lower compared to healthy controls, while infiltration of T cell subpopulations increases. Mechanistic studies show that IH upregulates TGF-β1 and IL-10 in human CD14^+^ monocytes, indicating a phenotypic shift that inhibits NK cell activity (72). OSA is strongly linked to immune system changes, with T cell activation and imbalance being key factors. CPAP treatment shows some reversing effects on these immune abnormalities.

### 3.6 Pattern recognition receptors phenotypes (TLR pathway)

Toll-like receptors (TLRs), as key components of the innate immune system, are crucial for recognizing pathogens and for initiating and sustaining systemic inflammatory cascade. Inhibiting TLR function can attenuate pro-inflammatory responses. OSA patients often exhibit concurrent upregulation of TLR4 and NF-κB, which together form the core axis of chronic IH-induced inflammatory responses (73–77). TLR4, as a typical pattern recognition receptor, can recognize pathogen signals or damage-associated molecular patterns, and through the adaptor protein MyD88, initiates a signaling cascade that rapidly activates NF-κB, leading to the expression of diverse inflammatory mediators (78, 79). In OSA patients, TLR2/6 expression is upregulated on immune cells, and this change correlates with the AHI. This may be explained by increased TLR2 promoter methylation and TLR6 gene body methylation accompanied by elevated protein expression. Chronic IH *in vitro* also induces upregulation of TLR2/6. These changes can be reversed by CPAP treatment (80, 81).

### 3.7 NLRP3 inflammasome

In severe OSA, NLRP3 activity in monocytes is elevated and positively correlates with the AHI and the hypoxia index. Under IH conditions, oxLDL or patient plasma can synergistically enhance NLRP3 activation and IL-1β production. Animal models show that genetic or pharmacological inhibition can alleviate brain, cardiovascular inflammation, and oxidative stress (18–22).

### 3.8 Immune checkpoint (PD-1/PD-L1)

In OSA patients, PD-L1 expression on peripheral monocytes and PD-1 expression on CD8^+^ T cells are elevated. The upregulation of PD-L1 transcription is mediated by HIF-1α, as detailed in section 2.1.3. This axis suggests a potential relevance to immunotherapy (27, 28).

The key points are summarized in **Table 1**.

**Table 1:** Immune cell dysregulation and regulation in OSA.

Immune Types/Targets	Major Abnormalities/Changes	Key Molecules or Pathways	Functional-Clinical Consequences	Reversibility/Intervention Evidence	Type of evidence	Ref
					clinical/preclinical data	
Neutrophils(NLR)	NLR↑ (positively correlated with OSA severity);	NLR, neutrophil activation, degranulation and activation increased	Promotes systemic inflammation, increased risk of coronary artery disease	NLR decreased after CPAP treatment	Clinical data	(47–49)
Monocyte-Macrophage	Macrophage count and activity ↑; M1 polarization ↑, ROS production ↑	HIF-1α, MIF/CD74, NF-κB	Enhanced pro-inflammatory response, increased risk of atherosclerosis, atrial fibrillation, and other conditions	Macrophage depletion/CPAP reversible	Mouse and rat model, *in vitro* data	(55–59)
Dendritic Cells (mDC/pDC)	Some studies show a decrease in total DCs; other studies found no significant difference	IL-6	Inhibition of T cell activation, immune damage	Mechanism and intervention pending	Clinical data	(24, 60)
γδ T cells	CD3^+^γδT cells are decreased in OSA patients; in hypoxia animal models, it first increases and then decreases		Early immune surveillance impairment	Data scarcity	Clinical data	(61)
NKT/iNKT	NKT-like cells ↑; iNKT cells ↓ and negatively correlated with AHI		Decline in anti-tumor immunity	CPAP can partially restore iNKT cell count and function	Clinical data	(62, 63)
CD4^+^/CD8^+^ T cells	Total count ↑; CD8^+^ activation (CD56/CD16, Perforin, TNF-α ↑); Th1↔Th2 imbalance	PSGL-1, CD40L, HIF-1α	Tissue damage, reduced immune surveillance	CPAP can reduce activation phenotype	Clinical data	(64–69)
B cells	Both the proportion and absolute number ↓	Undetermined (Obesity-metabolism related)	Amplification of systemic inflammation	Lack of intervention studies	Clinical data	(62)
NK cells	Count ↓, IFN-γ ↓	TGF-β1, IL-10	Impaired antiviral/antitumor immunity	Mechanistic studies suggest that inhibition is reversible	Clinical data	(49, 70–72)
TLR Pathway	TLR4/NF-κB synergistically upregulated; TLR2/6 methylation increased	TLR2/4/6, NF-κB, MyD88	Chronic inflammation	CPAP can downregulate TLR2/6	Clinical data, mouse and rat model,	(73–81)
NLRP3 Inflammasome	NLRP3 activity ↑, positively correlated with AHI, and synergistically activated with oxLDL	IL-1β, IL-18, ROS	Atherosclerosis, cerebral and cardiac injury	Pharmacological inhibition or gene knockout can alleviate	Clinical data, mouse and rat model, *in vitro* data	(17–22)
PD-1/PD-L1	Peripheral PD-L1 ↑, CD8^+^ T PD-1 ↑; HIF-1α directly upregulated	HIF-1α-PD-L1	Inhibition of T cells, immune escape, inflammation regulation	PD-1/PD-L1 inhibitors have potential therapeutic efficacy	Clinical data, mouse model, *in vitro* data	(26–28)

NLR, neutrophil-to-lymphocyte ratio; MDSCs, myeloid-derived suppressor cells; DCs, dendritic cells; mDC/pDC, myeloid/plasmacytoid DC; NK, natural killer; iNKT, invariant natural killer T cell; Th, T helper; Treg, regulatory T cell; TLR, Toll-like receptor; SF, sleep fragmentation; AHI, apnea-hypopnea index; CPAP, continuous positive airway pressure; oxLDL, oxidized low-density lipoprotein; PSGL-1, P-selectin glycoprotein ligand-1; CD40L, CD40 ligand; TGF-β 1, transforming growth factor-beta 1; MIF, macrophage migration inhibitory factor. Evidence types — Clinical: human studies; Preclinical: animal or *in vitro* studies; Both: both human and preclinical evidence.

## 4 Comorbidities associated with immune cluster changes in OSA

### 4.1 Cardiovascular diseases in OSA

Atherosclerosis is a chronic inflammatory condition caused by lipid metabolism disorders and abnormal adaptive immune responses (82). OSA triggers the following pathological cascade through IH:

IH first activates inflammatory pathways, directly damaging the vascular endothelium and contributing to OSA-associated hypertension and atherosclerosis (83). At the same time, sympathetic nervous system activation increases blood pressure and heart rate (84), promotes the extravasation of bone marrow progenitor cells, monocytosis, and upregulates inflammatory factors, further exacerbating endothelial dysfunction, amplifying systemic inflammation, and accelerating the formation and progression of atherosclerotic plaques (85).

In OSA-related immune dysregulation, multiple cellular pathways synergistically trigger vascular pathology: first, exosome-mediated intercellular communication enhances immune activation—B cells release exosomes carrying MHC-II-peptide complexes that directly stimulate CD4^+^ T cells (86); cardiovascular-associated cells, such as platelets, red blood cells, endothelial cells, monocytes/macrophages, and smooth muscle cells, release extracellular vesicles (EVs) that remodel macrophage phenotypes and inflammatory secretion, playing a continuous role in the development of atherosclerosis and hypertension (87–89).

Monocyte-macrophage system dysregulation constitutes the core pathological basis of vascular damage, where the signaling exchange between endothelial cells and monocytes/macrophages is not only a key link in maintaining cardiovascular homeostasis but also a core regulatory mechanism driving atherosclerosis (90). Specifically, IH mediates immune imbalance through dual pathways: 1) IH upregulates IL-6, driving macrophages to infiltrate adipose tissue and polarize to the pro-inflammatory M1 phenotype, inducing adipose inflammation and exacerbating insulin resistance and atherosclerosis (91); 2) IH simultaneously enhances CCR5 expression on monocytes, increasing their endothelial adhesion and chemotaxis to accelerate the progression of atherosclerosis (92). Meanwhile, T cell profile imbalance further aggravates the pathology—OSA patients show a significant increase in Th17 cell proportions, indicating that this subset plays a pathogenic role in inflammation-driven atherosclerosis (93). Ultimately, exosome-mediated immune activation, monocyte-macrophage dysregulation, and Th17 immune skewing collaboratively form the immunological network underlying OSA-IH-induced vascular damage.

Additionally, OSA exacerbates vascular damage by synergistically amplifying inflammatory signaling pathways: under sleep deprivation and IH, TLRs on macrophages are activated, inducing the release of pro-inflammatory cytokines and chemokines like TNF-α, IL-1, and IL-6, which cause endothelial dysfunction and initiate atherosclerosis (94). Clinical histological evidence shows that in moderate to severe OSA patients, TLR2, TLR4, TLR9, RAGE are elevated in carotid plaques, further emphasizing the critical role of the TLR-RAGE axis in OSA-related plaque formation (95). At the same time, the persistent activation of NF-κB upregulates miR-155 and miR-210, driving NLRP3 inflammasome formation and triggering inflammatory cascades, aggravating myocardial damage and vascular dysfunction (96).

**Figure 3:**
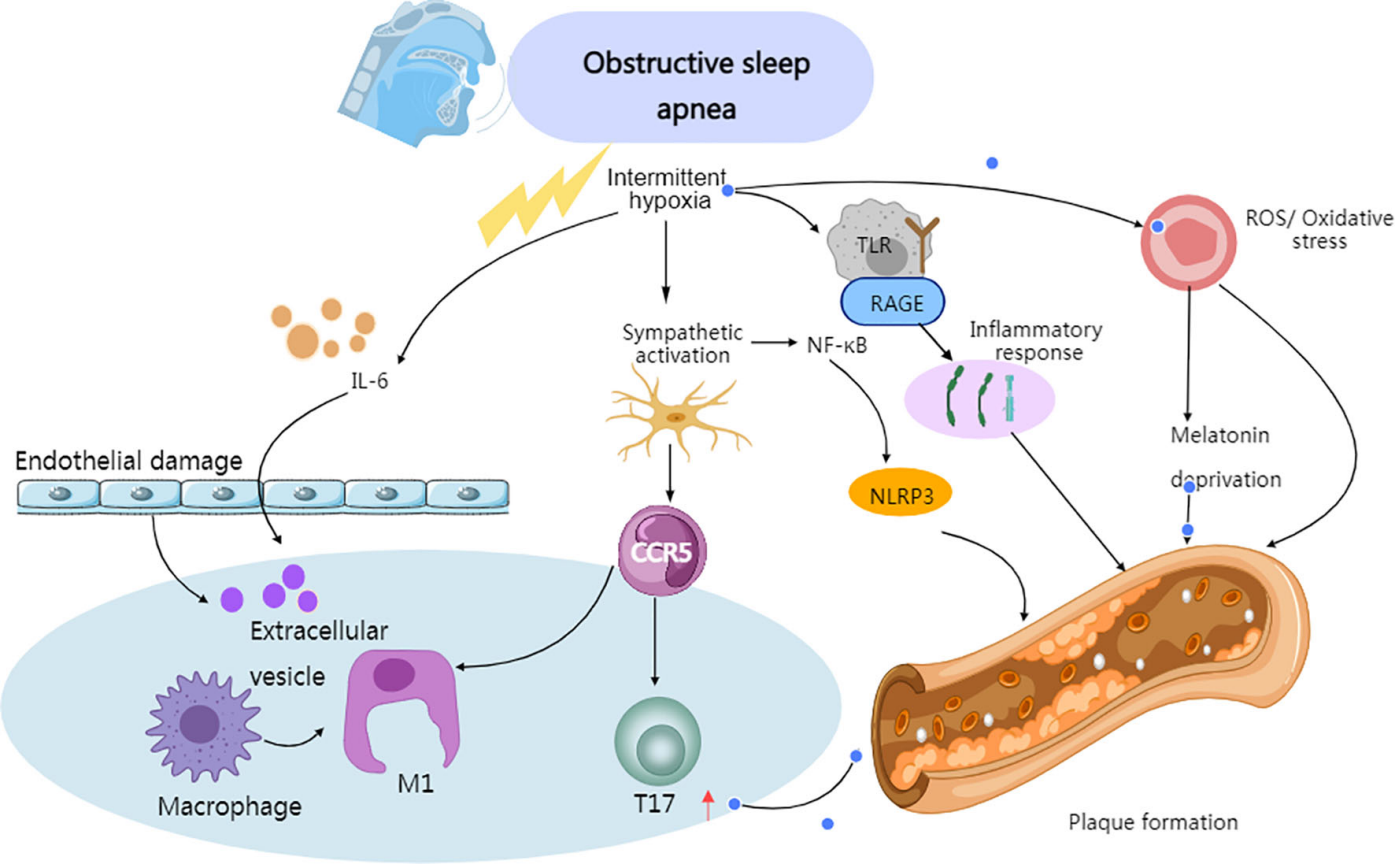
Immune-related mechanisms of atherosclerotic plaque formation induced by OSA. Created with MedPeer (medpeer.cn). Icons adapted and used under an institutional license for academic publication.

Oxidative stress is closely associated with the loss of protective mechanisms, with melatonin playing a protective role by reducing oxidative stress and the generation of inflammatory factors in immune and vascular cells, as well as inhibiting the progression of atherosclerosis. Therefore, the decrease in melatonin levels caused by sleep deprivation weakens its antioxidative, anti-inflammatory, and anti-atherosclerotic effects, which may be a potential mechanism for inducing vascular damage (97). Further *in vivo* evidence shows that the diversity of macrophages in arterial plaques is highly correlated with their continued exposure to lipids and their oxidized derivatives (98) (**Figure 3**).

For clinicians, several practical insights emerge from current evidence. First, circulating exosome profiles (particularly B-cell–derived exosomes and EVs from vascular cells) and altered monocyte/macrophage phenotypes may serve as candidate biomarkers for early detection of OSA-associated atherosclerosis. Second, elevated Th17/Treg imbalance and upregulation of inflammatory mediators (e.g., IL-6, TNF-α, CCR5 expression) could provide threshold indicators for initiating early anti-inflammatory or immunomodulatory interventions. Finally, reduced melatonin levels in OSA patients highlight the potential value of adjunctive antioxidant strategies in mitigating vascular injury.

### 4.2 Metabolic dysfunction in OSA

OSA patients are often at increased risk for metabolic issues like insulin resistance, type 2 diabetes, and dyslipidemia. Studies have shown that their adipose tissue exhibits inflammatory responses and functional impairments (99–102). It is important to emphasize that both IH and SF can independently contribute to metabolic disorders. Short-term exposure to IH has been found to decrease insulin sensitivity in healthy individuals (103, 104). In mouse models, although chronic hypoxia leads to weight gain, IH induces weight loss; however, both exacerbate visceral white adipose tissue (VWAT) inflammation and trigger metabolic disorders and insulin resistance. VWAT activates macrophages and spreads inflammatory signals through adipocyte-derived exosomes, which are key effectors in this pathological chain (105). Furthermore, substantial evidence from both human and rodent models consistently suggests that IH intervention disrupts systemic metabolic balance, but its specific mechanisms and key metabolic organs remain unclear (106–110). This also partly explains why OSA prevalence is higher in individuals with type 2 diabetes and hyperlipidemia (110). Notably, macrophage-derived extracellular vesicles (EVs) play a crucial regulatory role in innate immunity and can mediate inflammatory responses in specific metabolic tissues like VWAT. Under normal physiological conditions, EVs secreted by phagocytes are the major component of circulating EVs and carry molecular markers with protective effects against insulin resistance (IR). However, when the secretory cells are exposed to the abnormal environment caused by OSA, these protective features change, triggering IR within VWAT. In obese individuals with impaired metabolic function, VWAT typically shows extensive infiltration of immune cells such as macrophages (110, 111). The proportion and absolute number of pro-inflammatory M1 macrophages are significantly elevated, further exacerbating adipose tissue inflammation (112). Studies also indicate that exosomes released from adipocytes in obese individuals can activate tissue-resident macrophages in adipose tissue and induce them to secrete pro-inflammatory cytokines, thereby promoting insulin resistance (113). Existing evidence suggests that metabolic disorders and insulin resistance disrupt the balance between pro-inflammatory and anti-inflammatory mediators in macrophages, initiating a positive feedback loop that intensifies inflammatory macrophage activation and ultimately further impairs adipocyte function (110).

From a clinical perspective, several practical points should be considered. First, circulating extracellular vesicles—particularly macrophage- or adipocyte-derived EVs—may serve as emerging biomarkers for early detection of insulin resistance and adipose inflammation in OSA patients. Second, monitoring the M1/M2 macrophage ratio in visceral adipose tissue could provide a threshold indicator of metabolic deterioration and help stratify patients at higher risk of diabetes. Finally, recognizing OSA-related declines in insulin sensitivity even in non-obese individuals highlights the importance of early screening and timely initiation of metabolic interventions in this population.

### 4.3 Cognitive impairment associated with OSA

Several studies have clearly identified cognitive impairment as one of the major complications of OSA (114), and the cognitive decline process in OSA patients shares certain similarities with the pathogenesis of Alzheimer’s disease (AD) (115). Notably, OSA is more common in patients with cognitive impairment (116). In OSA patients, peripheral inflammation can trigger central nervous system inflammation by disrupting the blood-brain barrier or transmitting via the vagus nerve (117). Subsequently, infiltrating neutrophils form extracellular traps (NETs), further damaging the blood-brain barrier and activating microglial cells, ultimately impairing neurocognitive function (118). Additionally, IH promotes the damage of mitochondria, the release of mtROS and mtDNA, facilitates NLRP3 inflammasome assembly, activates caspase-1 and IL-1β release, and accumulates pro-inflammatory cytokines, which produce more mtROS, thereby triggering neuronal apoptosis and impairing hippocampus-dependent learning and memory functions (20). Another study also found that neuroinflammation triggers autophagy-lysosomal dysfunction, activating the NLRP3-caspase-1 inflammasome in the hippocampus of mice and BV2 cells, closely related to neuronal damage (119). Continuous positive airway pressure (CPAP), the primary treatment for OSA, can rapidly improve blood oxygen saturation and cognitive function (120). After short-term CPAP treatment, functional MRI showed improvements in memory and attention in OSA patients, associated with changes in the cerebellar cortex and bilateral hippocampus (121, 122). Therefore, existing data suggest that by improving oxygen saturation and regulating hippocampal function, CPAP treatment can effectively alleviate cognitive impairment in OSA patients. Therefore, Peripheral inflammatory markers (e.g., NETs, NLRP3 activation products) may serve as candidate biomarkers for early cognitive decline in OSA. Monitoring hippocampal function via imaging could guide timely interventions. Early initiation of CPAP remains the most effective strategy to prevent or reverse neurocognitive impairment.

### 4.4 Cancer immune editing in OSA

Recent studies suggest that OSA may contribute to the development and progression of various solid tumors through mechanisms like IH, oxidative stress, immune dysregulation, and remodeling of the inflammatory microenvironment. Epidemiological data indicate a significantly higher risk of colorectal cancer in OSA patients: a prospective study in Korea found that the detection rate of high-grade colorectal tumors in OSA patients was 3.03 times that of the control group, even after adjusting for age, gender, BMI, and smoking (123). A cohort study in Taiwan also showed that the risk in this population was 1.8 times higher than in non-OSA individuals (124). Common risk factors for both OSA and cancer, such as obesity and chronic inflammation, further strengthen the potential link between the two (125). Meta-analyses have confirmed that OSA is significantly linked to the incidence of prostate, breast, lung, and colorectal cancer (126, 127). Focusing on lung cancer, several studies have shown that OSA patients experience a higher occurrence of lung cancer during follow-up, with severe OSA significantly increases the mortality risk in late-stage patients (128, 129). Kendzerska et al. confirmed that OSA-related hypoxia indicators (such as AHI and average SaO_2_) are independent risk factors for lung cancer (130), while Seijo and Justeau found that T90% is a stronger predictor than AHI (HR = 2.14, 95% CI 1.01-4.54) (131, 132). Notably, the prevalence of OSA is also higher in lung cancer patients (133, 134), although some studies have reported that OSA may be negatively correlated with lung cancer in certain populations (135, 136). This suggests that the effect may be influenced by confounding factors such as age, sex, tumor type, and follow-up duration.

In terms of mechanism, animal experiments have found that IH stimulates tumor growth in non-small cell lung cancer (NSCLC) mouse models (137), but evidence on whether OSA affects lung cancer prognosis is insufficient (138). The synergistic impact of IH and SF may accelerate the occurrence and progression of lung cancer and promote treatment resistance through pathways like oxidative stress, chronic inflammation, immune dysfunction, and neuroendocrine disruption (139, 140). However, there is currently a lack of animal models that can accurately simulate both the IH and SF states in OSA patients, and the standards for IH exposure and SF quantification methods are not unified, limiting further research into the mechanisms. Research has demonstrated that chronic inflammation is strongly linked to the development of various cancers, with the immune system playing a crucial role in tumor angiogenesis, invasion, and metastasis. Regulatory T cells (Tregs), MDSCs, and tumor-associated macrophages (TAMs) can promote tumor progression. The repeated IH and tissue hypoxia caused by OSA can create a pro-cancerous environment by stabilizing HIF, promoting tumor angiogenesis and proliferation, disrupting immune surveillance, and enhancing immune escape. Clinical studies have found that pro-tumor gene expression is upregulated in peripheral white blood cells of OSA patients, which can be partially reversed after CPAP treatment (141).

In cancers associated with OSA, the PD-1/PD-L1 pathway is critically involved in facilitating immune evasion by tumors. In both clinical OSA cases and animal models, there is an upregulation of PD-1/PD-L1 expression, along with CD8^+^ T cell dysfunction and a higher proportion of MDSCs (142). A mouse model of OSA combined with NSCLC created by Huang et al. confirmed that PD-L1 expression is closely related to the intensity of IH, and tumor burden increases in a PD-L1-dependent manner (143). Further studies show that IH can drive PD-L1 overexpression through the upregulation of HIF-1α (144, 145); IH increases HIF-1α and PD-L1 in tumor cells, weakening cytotoxic T cells and increasing TAMs, thus accelerating tumor progression (146). Additionally, exosomes released by lung cancer cells have been found to upregulate PD-L1 expression on TAMs (147). Clinical studies show that the levels of soluble PD-L1 (sPD-L1) in the serum of severe OSA patients are significantly elevated (148). Since elevated PD-L1 levels are closely linked to enhanced immune evasion by tumors, targeting the PD-1/PD-L1 axis may represent a promising treatment strategy for individuals with lung cancer who also suffer from OSA.

IH promotes the polarization of TAMs towards the M2 subtype, which is known to support tumor progression and facilitate cancer cell invasion. Animal experiments have confirmed that IH accelerates lung cancer progression, and cyclooxygenase-2 (COX-2) inhibitors can block M2 polarization of TAMs, delaying tumor deterioration (149–151). At the same time, IH induces tumor cells to secrete interleukin-10 (IL-10), which in turn facilitates the polarization of TAMs towards the M2 phenotype (152), and also promotes the enrichment of immunosuppressive cell populations, including MDSCs, granulocytes, and Tregs, together creating a pro-tumor immune-suppressive microenvironment (153).

Transforming growth factor-beta (TGF-β), a core regulator of the tumor immune microenvironment, can regulate tumor proliferation, invasion, and microenvironment remodeling (154). Studies have shown that in untreated OSA patients, monocytes release TGF-β to suppress NK cell function (which can be restored with CPAP treatment) (72), and IH can activate the TGF-β pathway to promote lung cancer cell migration and activation of cancer-associated fibroblasts (CAFs) (155). In an OSA combined NSCLC model created by Akbarpour et al., IH and SF significantly weakened the tumor-killing effect of cytotoxic T lymphocytes (CTLs), promoting cancer stem cell (CSC) immune evasion and maintaining self-renewal (156).

iNKT, important anti-tumor immune cells, are impaired in both number and function in severe OSA patients, and CPAP treatment can partially restore their function (63, 114), suggesting that iNKT dysfunction may also participate in the immune escape mechanism of OSA-related tumors. Zhang et al. found that IH can increase tumor volume and weight, while upregulating the expression of vascular endothelial growth factor (VEGF) and endothelin-1, enhancing angiogenesis (157). Kang et al. provided further evidence that in a lung adenocarcinoma mouse model exposed to IH, VEGF levels were significantly elevated, and its regulation was mediated primarily by nuclear factor erythroid 2-related factor 2 (Nrf2) and β-catenin, rather than HIF-1α (158). Additionally, in the IH combined with non-small cell lung cancer (NSCLC) model, the expression of ATAD2 (ATPase family protein) was significantly increased. Knockdown of ATAD2 inhibited lung cancer cell invasion and migration, reduced mtROS production, and decreased both the quantity and functional capacity of CSCs, suggesting that IH accelerates lung cancer progression by activating the HIF-1α/ATAD2 axis, regulating mtROS and CSC interactions (159).

**Figure 4:**
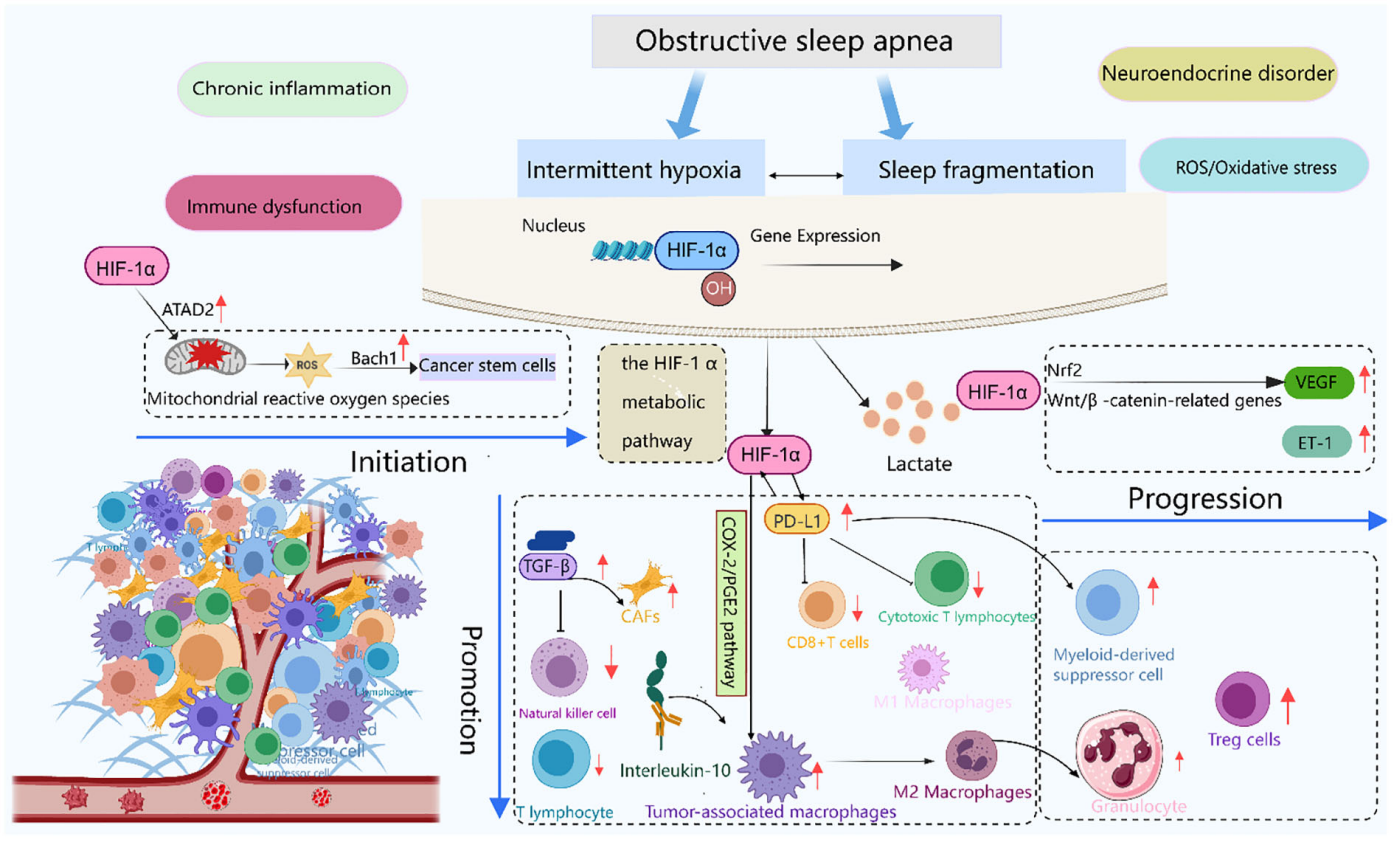
Immune stress response induced by OSA and its role in tumor evolution. This schematic illustrates the temporal sequence of tumor progression under OSA-induced immune stressors. Initiation: Intermittent hypoxia stabilizes HIF-1α, acting as the triggering molecular event. Promotion: HIF-1α signaling upregulates PD-L1 expression and drives TAMs toward M2 polarization, establishing an immunosuppressive microenvironment. Progression: VEGF-mediated angiogenesis, together with sustained immune evasion, facilitates tumor invasion and metastasis. Together, these steps outline the continuum from OSA-driven immune dysregulation to cancer progression. Created with MedPeer (medpeer.cn). Icons adapted and used under an institutional license for academic publication.

Current research confirms that IH can trigger secondary inflammation, oxidative stress-induced cellular injury, immune system impairment, and alterations in tumor-related genes. Concurrently, IH elevates the expression of key signaling molecules, including PD-L1 and VEGF. It also promotes the expansion and specialization of TAMs, CSCs, and vascular endothelial cells. Furthermore, IH mediates intercellular communication via exosome signaling. Collectively, these mechanisms facilitate tumor cell proliferation, increase invasiveness, stimulate neovascularization, and accelerate malignant progression. However, there are still limitations in current research: first, there is currently no universally accepted animal model that can simultaneously simulate the core features of IH and SF, and studies on SF are relatively insufficient; second, the majority of *in vivo* and *in vitro* studies primarily concentrate on the roles of IH in enhancing tumor cell growth and invasiveness, without in-depth analysis of its effects on distant metastasis, and lack verification of the carcinogenic potential of IH; third, cancer development involves multiple mechanisms including redox imbalance, persistent inflammatory responses, immune evasion, and disturbances in neuroendocrine regulation, and there is still a lack of systematic research integrating these key mechanisms(**Figure 4**). We believe that serum soluble PD-L1 and VEGF may serve as candidate biomarkers for OSA-related tumor progression. Monitoring Treg/MDSC/TAM profiles could help identify patients at higher oncologic risk. Early CPAP intervention and potential PD-1/PD-L1–targeted therapies may mitigate cancer immune escape in OSA.

### 4.5 Autoimmune diseases in OSA

Th17 cells, as a critical effector population mediating cellular immunity, are essential in the development of autoimmune and allergic conditions, whereas Tregs function to preserve immune system tolerance, preventing organ-specific autoimmunity, allergies, and transplant rejection. The dynamic balance between these two cell types is a core mechanism regulating immune homeostasis and modulating inflammation. Research has revealed that in OSA patients, cytokines promoting Th17 differentiation, such as IL-6 and IL-17, are markedly increased, while TGF-β1 levels are decreased, creating an inflammatory cytokine-dominated immune microenvironment, thus disrupting the Th17/Treg balance (160, 161). Domestic studies have also found that the Th17/Treg ratio in individuals with OSA is significantly higher than in healthy individuals, and this ratio shows a positive association with both the severity of OSA and C-reactive protein levels (162), suggesting that it may be involved in the autoimmune pathological process related to OSA. Furthermore, some researchers have proposed that OSA may act as one of the triggers of autoimmune responses: repeated IH can cause cellular damage, leading to hyperuricemia and affecting the maturation and antigen-presenting function of dendritic cells. When sodium urate crystals accumulate to a certain extent, they can activate T cell responses, disrupting immune tolerance and promoting the initiation and advancement of autoimmune disorders (163). Based on existing evidence, the Th17/Treg ratio may represent a candidate biomarker for assessing OSA-related autoimmune risk. Elevated IL-6 and IL-17, together with reduced TGF-β1, could serve as potential thresholds for early immunomodulatory intervention. In addition, monitoring uric acid levels may help identify patients at risk of autoimmunity triggered by OSA.

## 5 Discussion and future perspectives

This review comprehensively highlights the significant influence of OSA on immune function and explains its pathological connections with multiple comorbidities across molecular, cellular, and organ systems. Overall, repeated IH and SF constitute the two core immune stressors of OSA. The former amplifies oxidative stress and inflammatory responses through the ROS-HIF-1-NF-κB-NLRP3 axis, while the latter further weakens immune homeostasis via multiple pathways, including circadian disruption, loss of slow-wave sleep, excessive sympathetic activation, and Th1/Th2 switching. Together, these factors synergistically drive the shared immune foundation for atherosclerosis, insulin resistance, neurodegenerative changes, and tumorigenesis.

At the cellular level, MDSC expansion, M1 macrophage polarization, variable dendritic cell function, and γδT/iNKT defects form the innate-adaptive immune network. Notably, T cell profile dysregulation deserves attention: (1) CD8^+^ cytotoxic T cells become excessively activated due to upregulation of NK receptors and perforin/TNF-α, exacerbating tissue damage; (2) CD4^+^ subsets exhibit plasticity in Th1↔Th2 oscillation, suggesting susceptibility to regulation by metabolic and neuroendocrine backgrounds; (3)The elevated Th17/Treg ratio is highly correlated with autoimmune and vascular wall inflammation. Meanwhile, HIF-1α enhances the expression of the PD-1/PD-L1 pathway under OSA conditions, resulting in T cell dysfunction and facilitating tumor evasion from immune surveillance.

Clinical evidence indicates that CPAP can partially reverse early immune activation: it reduces NLR, restores γδT/iNKT function, inhibits CD8^+^ NK-like phenotypes, and downregulates PD-L1. However, efficacy is highly dependent on treatment adherence and duration, with limited improvement in established immune memory or organ damage. Notably, Beyond CPAP, several alternative and adjunctive interventions may mitigate OSA-related immune dysregulation through distinct biological mechanisms. Hypoglossal nerve stimulation (HNS) and other upper-airway neuromodulation techniques enhance airway patency and sleep architecture, thereby alleviating IH and SF. By reducing hypoxic burden, these approaches are expected to suppress HIF-1α stabilization, ROS generation, and downstream activation of the NF-κB/NLRP3 pathway, resulting in lowered systemic inflammatory markers (e.g., NLR, IL-6) and partial restoration of T-cell and NK cell function. Targeted anti-inflammatory strategies—such as inhibitors of upstream mediators (e.g., NLRP3 or IL-1β), ROS scavengers, melatonin supplementation, and, in selected oncologic settings, modulation of the PD-1/PD-L1 immune checkpoint axis—represent mechanism-based approaches to interrupt IH-driven inflammation. However, clinical data in OSA populations remain limited, and the systemic safety profile, including infection risk, warrants careful evaluation. Lifestyle interventions and structured exercise programs (with or without weight loss) reduce visceral adiposity, downregulate pro-inflammatory cytokines, enhance insulin sensitivity, and promote anti-inflammatory myeloid cell phenotypes, thereby addressing a key modifiable factor in OSA-related immune abnormalities. Finally, combination strategies—such as device-based therapies integrated with anti-inflammatory or metabolic treatments—may offer enhanced benefit for patients with persistent immune activation despite adequate airway management. Well-designed randomized controlled trials incorporating predefined immunologic endpoints and standardized adherence metrics are essential to elucidate the magnitude, durability, and clinical significance of immunomodulation achieved through these interventions.

In conclusion, OSA is not only a sleep-disordered breathing condition but also a “systemic immune disease.” Decoding its immune code, precisely blocking key pathways, and synergizing with traditional therapies may become a key breakthrough in reducing multi-organ damage and mortality burden in OSA patients.
